# Unexpected normal left ventricular systolic function after total chronic occlusion of left main coronary artery and stenosis of right coronary artery: a case report and review of the literature

**DOI:** 10.1186/s13256-019-2310-6

**Published:** 2019-12-23

**Authors:** Younes Moutakiallah, Reda Mounir, Amir Aden Ali, Fouad Nya, Aniss Seghrouchni, Noureddine Atmani, Abdelmajid Bouzerda, Zouhair Lakhal, Mohamed Drissi, Mahdi Aithoussa

**Affiliations:** 1 Faculty of Medicine and Pharmacy, Mohammed V University, Rabat, Morocco; 2 Cardiac Surgery Department, Mohammed V Teaching Military Hospital, Rabat, Morocco; 3 Cardiology Department, Mohammed V Teaching Military Hospital, Rabat, Morocco; 4 Intensive Care of Cardiac Surgery, Mohammed V Teaching Military Hospital, Rabat, Morocco

**Keywords:** Total chronic occlusion, Left main coronary artery, Unexpected normal left ventricle, Coronary artery bypass grafting

## Abstract

**Introduction:**

Total occlusion of the left main coronary artery is a very rare finding in coronary angiography because of its highly lethal nature. Right coronary artery dominance and extensive collateral circulation are the principal determinant factors of survival after total occlusion of the left main coronary artery. The impact on the left ventricle is often significant with a profound alteration of its systolic function.

**Case presentation:**

We describe a 52-year-old North African man, a tobacco smoker, who presented symptoms of unstable angina related to a total chronic occlusion of his left main coronary artery with a right coronary artery stenosis. Unexpectedly, the impact on his left ventricle was absent with normal dimensions and systolic function. He underwent a successful on-pump coronary artery bypass grafting with uneventful postoperative course and good recovery.

**Conclusions:**

Total occlusion of the left main coronary artery is a rare condition, the fact that the left ventricle retains a normal size and systolic function makes it exceptional, which must be kept in mind to avoid dangerous examinations and delayed treatment. Coronary artery bypass surgery should be considered the main treatment of total chronic occlusion of the left main coronary artery.

## Introduction

Left main coronary artery (LMCA) disease is largely dominated by atheromatous stenosis with variable scale of severity. The total occlusion of LMCA is still a surprising discovery in a cardiac catheterization laboratory with a very low incidence ranging from 0.04 to 0.43% in a reported series [1–9]. Unfortunately, sudden death is a frequent mode of revelation of this highly lethal pathology, which might minimize the true estimation of LMCA occlusion prevalence [7]. Extensive development of collateral circulation [5] and dominant right coronary artery (RCA) are the principal determinants of myocardial vascularization and clinical presentation. However, left ventricular (LV) function is usually depressed especially in cases of associated RCA stenosis [3, 4].

Myocardial revascularization, whether surgical or percutaneous, remains the best treatment of total chronic occlusion of the LMCA with a superiority of coronary artery bypass graft (CABG) over percutaneous coronary intervention (PCI) for complex lesions. We report the case of a patient who underwent on-pump CABG for total chronic occlusion of the LMCA.

## Case presentation

A 52-year-old North African man was transferred from an emergency unit to our department for unstable angina of 24 hours’ duration. He had a history of hypertension and dyslipidemia and he had smoked tobacco for 30 years. Progressive effort-induced angina and exertional dyspnea had been deliberately neglected for 10 years until recent onset of acute coronary syndrome. On clinical examination, he was hypertensive at 142/83 mmHg with a regular pulse at 59/minute and body mass index of 25 kg/m^2^. His heart sounds were normal without any additional sounds or murmurs. His lungs were clear to auscultation and there was no peripheral edema or jugular venous turgor. The auscultation of carotid arteries was normal.

**Fig. 1:**
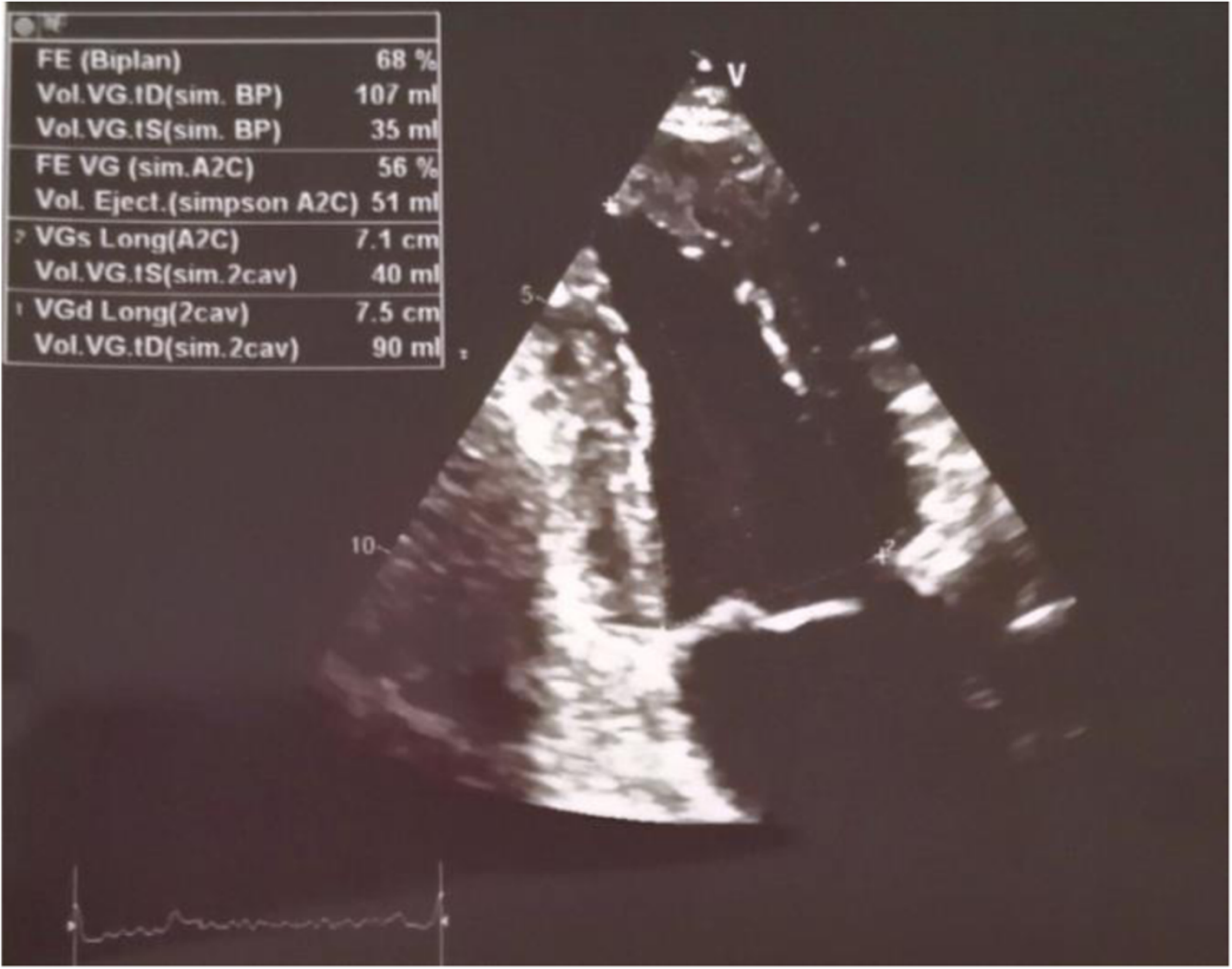
Transthoracic echocardiographic image showing the normal left ventricular size and function

**Fig. 2:**
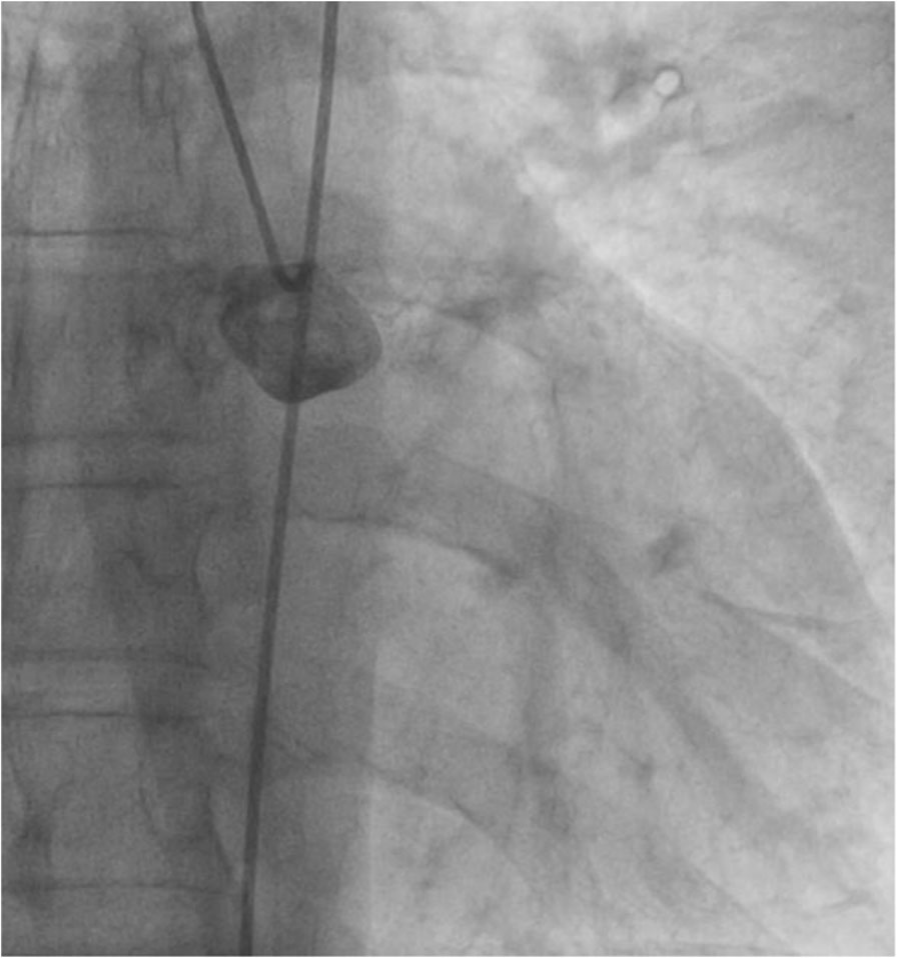
Coronary angiography showing no antegrade flow in the left main coronary artery and its collaterals. Notice the calcifications of the coronary artery wall

**Fig. 3:**
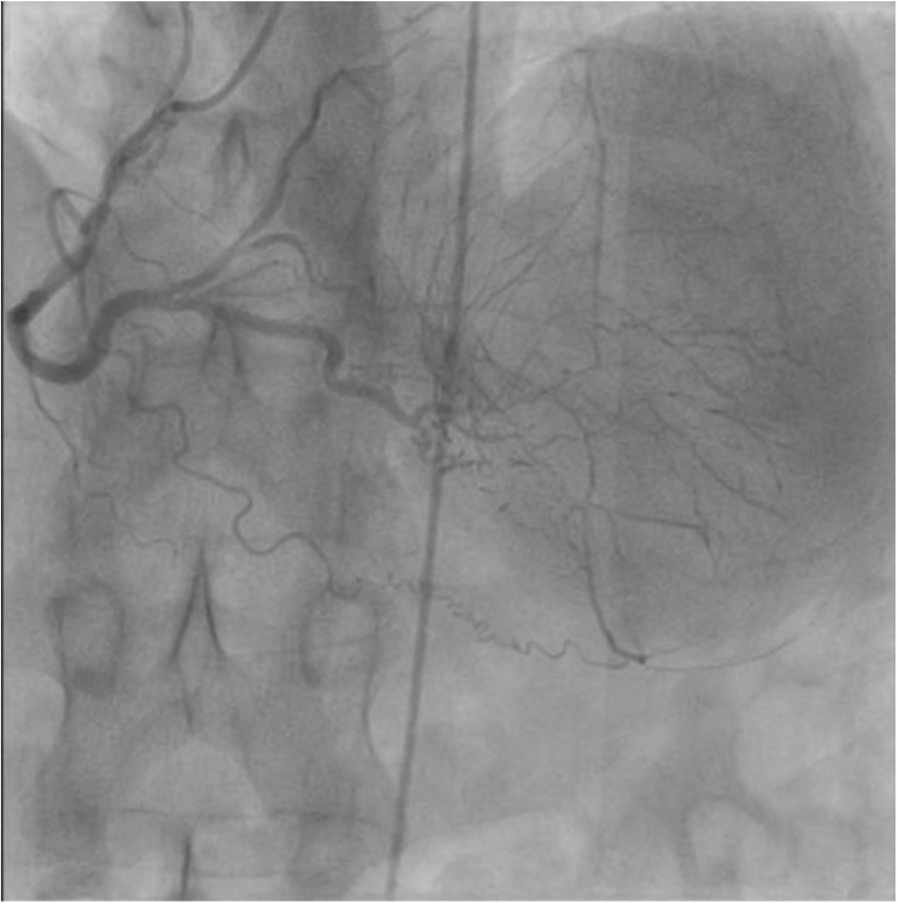
Coronary angiography showing the extensive and rich collateral circulation bonding right and left coronary systems

**Fig. 4:**
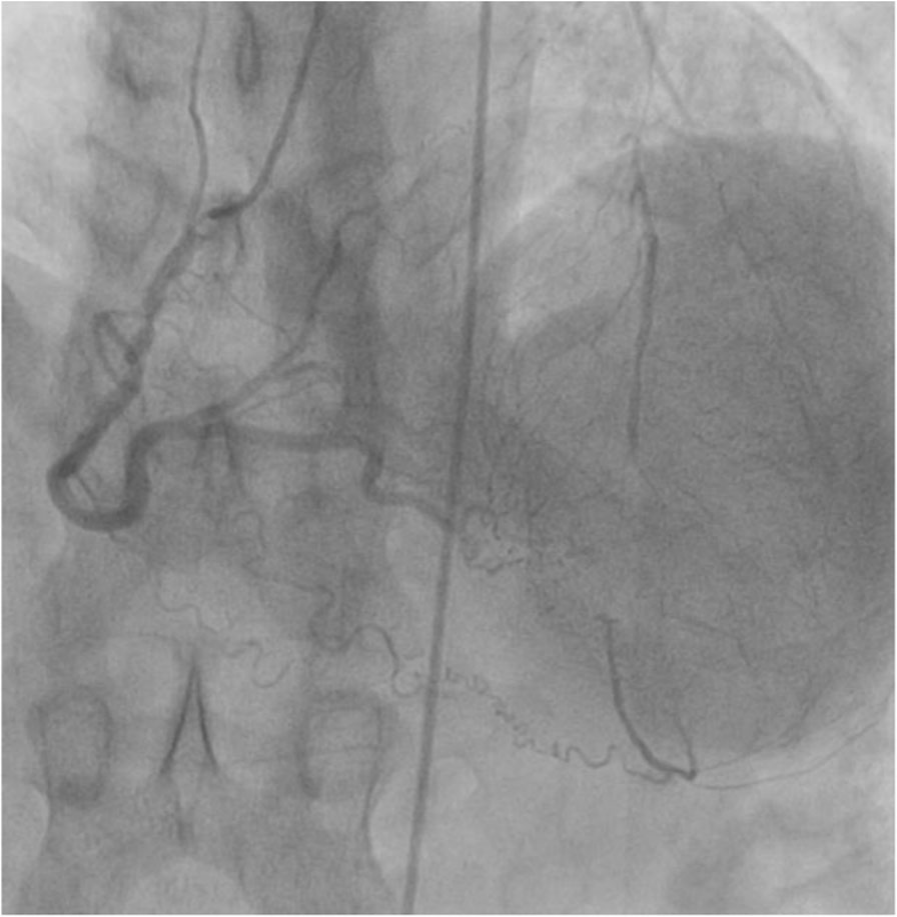
Coronary angiography showing the retrograde filling of left coronary system by collaterals issued from dominant right coronary artery. Notice the stenoses of the right coronary artery and the left anterior descending artery

Resting electrocardiogram showed normal sinus rhythm at 55 beats per minute (bpm) without any ST segment changes. There were anteroseptal Q waves and inverted T-waves on apical and lateral leads. A transthoracic echocardiogram showed a normal LV size and function (LV ejection fraction at 68%) with normal motions of all segments (Fig. 1). The LV filling pressures were low. Systolic pulmonary artery pressure was 24 mmHg and the right ventricular size and function were normal. Coronary angiography revealed total occlusion of LMCA with no antegrade flow in the left anterior descending artery (LAD) and the circumflex artery (Cx) (Fig. 2). RCA was dominant and well developed; it irrigated the left coronary arterial network through an extensive collateral circulation (Fig. 3). However, RCA had a significant stenosis (Fig. 4).

He was referred 2 days after coronary angiogram to surgery for elective on-pump CABG. Anesthesia was uneventful: cisatracurium besylate, midazolam, thiopental, and propofol. After median sternotomy, the internal thoracic arteries and the saphenous vein graft (SVG) were harvested. His right internal thoracic artery (RITA) was anastomosed to left internal thoracic artery (LITA) to make composite “LITA-RITA-Y” graft configuration. A cardiopulmonary bypass, performed by aortic and venous cannulation, was conducted under moderate systemic hypothermia. Myocardial protection was achieved with antegrade cold blood high potassium cardioplegia. We carried on with distal anastomoses of SVG to RCA, RITA to obtuse marginal branch, and, finally, LITA sequentially to diagonal artery and LAD. His postoperative course was uneventful and he was discharged at day 9. The 6-month control showed good recovery without residual chest pain or dyspnea and a normal physical examination without signs of heart failure. An electrocardiogram showed normal sinus rhythm at 63 bpm with no ST segment changes and persistence of anteroseptal Q waves, but disappearance of inverted T-waves on apical and lateral leads. An X-ray of his chest was normal without cardiomegaly; transthoracic echocardiography showed normal LV size and function with 62% of ejection fraction and normal segmental contractility.

## Discussion

Our observation had several specificities: first, total occlusion of LMCA is very rare; second, there was no parallelism between the severity of the pathology and the relatively non-threatening clinical presentation; third, the LV size and function were preserved despite an associated stenosis of a dominating RCA; and fourth, we report the surgery contribution to the therapeutic arsenal.

Total occlusion of LMCA is lethal usually by sudden cardiac death after massive myocardial infarction and cardiogenic shock [9, 10]. Samadov *et al.* suggested that the high probability of out-of-hospital cardiac arrest might preclude the true estimation of the prevalence of LMCA occlusion [7].

On clinical examination, the patients are often strongly symptomatic with complaints of recurrent typical chest pain, or a disabling exercise angina occurring at the slightest effort of daily life or even during rest. They may have a history of myocardial infarction or present symptoms of heart failure with advanced exertional dyspnea or even rest dyspnea [8]. According to Su *et al.,* cardiogenic shock has been described in LMCA occlusion, and timely mechanical support should be considered for emergency revascularization in such cases [11]. In our case, symptoms were less severe and even less suggestive of the seriousness of the pathology.

Since the main artery cannot ensure LV vascularization, two conditions are required for both patient survival and maintenance of LV function: a dominant RCA, and a massive and rich collateral circulation allowing retrograde vascularization of the entire left coronary network [5]. This notion of right dominance has been almost always apparent in the literature [7, 9, 11]. However, in cases of significant RCA stenosis, the LV size and function are usually altered because of the myocardial ischemia. Zimmern *et al.* showed that 50% of patients had more than 50% of RCA stenosis [2, 9]. Ipek *et al.* reviewed seven cases of total occlusion of LMCA and found that of the four patients who had an impaired LV function, three had a RCA stenosis [1]. This contrasts with our patient’s case who despite having a stenosis of the RCA, showed no abnormality in his LV. Su *et al.* estimated that LV function and myocardial salvage are accurately related to LMCA occlusion duration [11]. Shaikh *et al*. suggested that it is unlikely to find significant distal left coronary disease in cases of normal or nearly normal LV functions [9]. This contrasts with our case since our patient had normal LV function while having distal coronary lesions on both right and left coronary arteries. Shen *et al.* had identified, in a larger study of 35 patients treated by CABG for total occlusion of LMCA, the presence of insufficient collaterals and significant RCA stenosis as predictors of long-term decreased survival [6].

Coronary angiography assessment of left coronary arteries may be difficult to perform because of sluggish filling of the coronary arteries via collaterals [9]. In our case, the LAD had small caliber with an atheromatous and irregular wall and several significant stenoses, and the Cx had a significant proximal stenosis.

The treatment mainly involves myocardial revascularization techniques, namely CABG and PCI [4]. In our case, we opted for surgery for several reasons; we cite the young age of the patient, the complexity of the coronary lesions that were extensive, the high SYNTAX score which was 51, and the complete revascularization especially that we used the two internal thoracic arteries. De Rosa *et al*. demonstrated that similar outcomes are to be expected from stent-PCI and CABG in appropriately selected patients with significant LMCA disease, when performed by experienced teams. However, they concluded that there was a better performance for CABG in specific patients’ categories, such as more complex anatomical settings, whereas stent-PCI showed a better performance in older age groups and less extensive coronary vascular disease [12].

## Conclusions

Normal LV function does not exclude coronary artery disease as severe as total occlusion of LMCA, which should lead us to be more cautious about chronic signs of myocardial ischemia. Certainly, it is a surprising discovery that raises fears, but it should in no way be an obstacle for an elective and thoughtful myocardial revascularization procedure. A scheduled coronary artery bypass surgery with complete revascularization should be considered the first therapeutic indication.

## Data Availability

Please contact author for data requests.

## Abbreviations

bpm: Beats per minute
CABG: Coronary artery bypass graft
Cx: Circumflex artery
LAD: Left anterior descending artery
LITA: Left internal thoracic artery
LMCA: Left main coronary artery
LV: Left ventricle
PCI: Percutaneous coronary intervention
RCA: Right coronary artery
RITA: Right internal thoracic artery
SVG: Saphenous vein graft
